# Acute quadriplegia caused by necrotizing myopathy in a renal transplant recipient with severe pneumonia: acute onset and complete recovery

**DOI:** 10.1186/s40001-015-0087-7

**Published:** 2015-02-03

**Authors:** Guo-wei Tu, Jie-qiong Song, Simon Kang Seng Ting, Min-jie Ju, Hong-yu He, Ji-hong Dong, Zhe Luo

**Affiliations:** Department of Critical Care Medicine, Zhongshan Hospital, Fudan University, Shanghai, 200032 PR China; National Neuroscience Institute, Singapore General Hospital Campus, Singapore, Singapore; Department of Internal Neurology, Zhongshan Hospital, Fudan University, Shanghai, 200032 PR China

## Abstract

Critical illness polyneuropathy and myopathy are multifaceted complications that follow severe illnesses involving the sensorimotor axons and proximal skeletal muscles. These syndromes have rarely been reported among renal transplant recipients. In this paper, we report a case of acute quadriplegia caused by necrotizing myopathy in a renal transplant recipient with severe pneumonia. The muscle strength in the patient’s extremities improved gradually after four weeks of comprehensive treatment, and his daily life activities were normal a year after being discharged.

## Background

Critical illness polyneuropathy and myopathy (CIP/CIM) are complex syndromes caused by various pathophysiological mechanisms in critically ill patients. Muscle weakness and functional impairment can be self-limited for weeks to months. However, in certain severe cases, these symptoms can last up to five years after the patient is discharged [1,2]. No therapies have been proven effective. The majority of CIP/CIM cases have been reported in patients with multi-organ dysfunction and sepsis, and these syndromes have rarely been reported in renal transplant recipients [3]. In this paper, we present a case of extensive necrotizing myopathy with favorable recovery in a renal transplant recipient with severe pneumonia.

## Case presentation

Our patient was a 60-year-old man with a history of living-related renal transplantation and subsequent immunosuppression therapy (tacrolimus (6 mg/d) plus mycophenolate mofetil (2 g/d) plus prednisolone (10 mg/d)) for four months. He was admitted to the intensive care unit (ICU) with a five-day history of fever and hypoxia. Bilateral pulmonary inflammatory infiltration was observed via a chest computed tomography scan. The patient was empirically treated with moxifloxacin, ganciclovir and trimethoprim-sulfamethoxazole as anti-infective therapy. Caspofungin was later administered because a fungal infection was suspected. Finally, the presence of serum cytomegalovirus (CMV)-immunoglobulin M and CMV-DNA suggested a diagnosis of CMV pneumonia.

Along with antibiotics, we gave our patient methylprednisolone (60 mg) every 12 h to reduce the pulmonary inflammatory infiltration, and all immunosuppressants were discontinued [4]. He initially received non-invasive positive-pressure ventilation therapy. However, after 19 days, severe hypoxemia resulted in him undergoing endotracheal intubation and mechanical ventilation. Rapid sequence induction was performed using propofol (100 mg), fentanyl (0.05 mg) and rocuronium bromide (50 mg). A combination of propofol and morphine was administered continually for sedation and analgesia. ‘Daily wake-up’ (approximately 30 min to 2 h) was performed according to the routine protocol in our ICU. Our patient underwent a percutaneous dilatational tracheostomy three days later. Ten days after the tracheostomy, sedation was discontinued, and he was successfully weaned from the mechanical ventilator.

**Figure 1:**
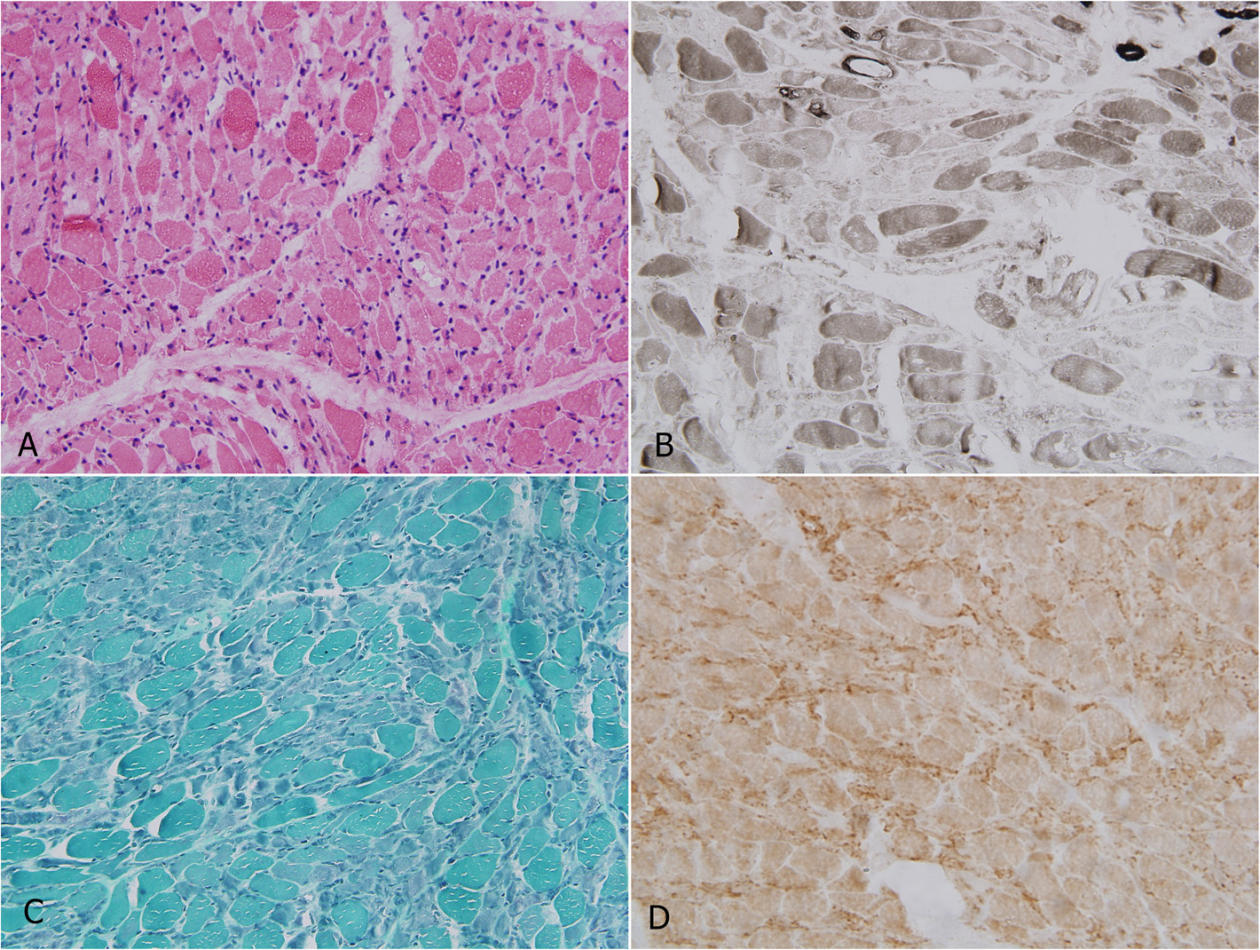
**Light microscopy of the rectus femoris muscle. (A)** Hematoxylin and eosin staining revealed extensive muscle fiber necrosis with prominent myofibrillar disarray. **(B)** ATPase (pH 9.4) reactions also revealed muscle fiber necrosis and decreased ATPase activity in type 2 fibers. **(C)** Modified Gomori trichrome staining revealed disarrayed muscle fibers without any ragged red fibers. **(D)** Oil red O staining revealed no heavy lipid deposition in the muscle fibers.

**Figure 2:**
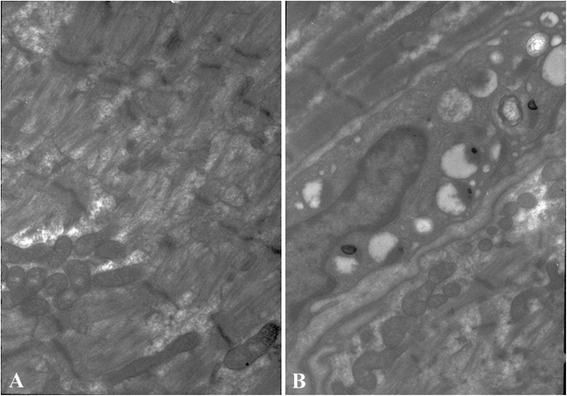
**Electron micrograph of the rectus femoris muscle. (A)** There were few thick myofilaments in the loose muscle, whereas Z discs were less affected. **(B)** Mitochondria with abnormal appearance, such as disorganization and reduction or disappearance of the cristae.

Our patient had severe, flaccid quadriplegia after his tracheostomy. A neurologic examination demonstrated bilateral flaccid weakness in his lower and upper extremities, which were Grade I according to the Medical Research Council (MRC). Generalized areflexia was present in all of his extremities. However, his sensory examination was unremarkable. A nerve conduction study demonstrated low-amplitude compound muscle action potentials (CMAPs) with increased durations in the bilateral median, ulnar, tibial and peroneal motor nerves. Results from sensory nerve action potential and repetitive nerve stimulation studies were normal. Needle electromyography revealed positive sharp waves and myogenic motor unit potentials in his biceps, deltoids and vastus medialis (bilaterally). A muscle biopsy from the right rectus femoris revealed extensive muscle fiber necrosis via hematoxylin and eosin staining. No obvious inflammatory infiltration was detected (Figure 1A). ATPase 9.4 staining was reduced or absent in the type 2 fibers (Figure 1B). Modified Gomori trichrome staining revealed disarrayed muscle fibers without any ragged red fibers (Figure 1C). No heavy lipid deposition was found in the muscle fibers via Oil red O staining (Figure 1D). Electron microscopy revealed prominent myofibrillar disarray and uniform-sized mitochondria (Figure 2). The serum level of creatine kinase was 180 U/L (range: 52 to 336 U/L).

Our patient was diagnosed with CIP/CIM. Adequate enteral nutritional support, B vitamins and coenzyme Q10 were administered immediately. Daily rehabilitation was also performed under the guidance of an occupational therapist. Methylprednisolone was reduced to 12 mg without additional immunosuppressant therapy. Two weeks later, the muscle strength in our patient’s extremities began to improve slowly and reached MRC Grade III after four weeks. A renal transplant biopsy revealed no evidence of acute rejection. His muscle strength improved steadily, and his activities of daily life were normal a year after he was discharged.

## Discussion

CIP/CIM, neuromuscular disorders that develop after admission to the ICU, are associated with prolonged mechanical ventilation and length of ICU stay, and increased hospital mortality [5]. The clinical risk factors for CIP/CIM include malnutrition, sepsis, a long duration of mechanical ventilation, immobility, hyperglycemia, and the use of glucocorticoids and neuromuscular blocking agents [6-9]. In the reported case, although our patient received only 50 mg of rocuronium bromide as rapid sequence induction, its side effects could not be excluded [10]. In addition, glucocorticoids given in combination with neuromuscular blocking agents increase the risk of neuromuscular weakness. Previous studies have suggested a high incidence of weakness in patients mechanically ventilated for more than four to seven days. As a result, 13 days of mechanical ventilation was also considered to be a risk factor of CIP/CIM in our patient.

The majority of CIP/CIM cases are diagnosed based on clinical manifestation and electromyography. The clinical characteristic of CIP/CIM is primary axonal degeneration (without demyelination), which typically affects motor nerves more than sensory nerves and results in symmetrical muscular atrophy [11]. The electrophysiological characteristics of these syndromes are reduced amplitudes and increased durations of CMAPs. In CIP, the amplitudes of the sensory nerve action potentials are also reduced [6]. Despite the invasive nature of histopathological and immunopathological approaches, they are considered to be the gold standard for diagnosing CIP/CIM [12]. The pathological features of CIM are classified into the following three main types: necrotizing, non-necrotizing, and selective loss of thick myosin myofilaments [13]. Some studies have reported a significant overlap between these pathophysiological processes in critically ill patients [14]. The typical manifestations of the patient reported in this paper were muscle weakness, areflexia, myogenic damage observed via nerve conduction and electromyographic study, and extensive necrotizing myopathy observed via muscle biopsy. To the best of our knowledge, this paper is the first report of CIM with necrotizing myopathy in a renal transplant recipient following severe pneumonia.

The main differential diagnosis in our patient was steroid myopathy, which presented with a normal serum cytokeratin level and muscular atrophy mainly involving the proximal part of his lower limb. Unfortunately, we selected the rectus femoris of our patient as the site for an open muscle biopsy because of the severe muscular atrophy in the proximal and distal parts of all four extremities. Although the myogenic changes in the needle electromyography and the type 2 fiber atrophy found in steroid myopathy are similar to those in CIM, such changes usually occur after several weeks or months after steroid administration. Our patient had a more rapid onset than those reported in steroid myopathy [15].

The management of our patient principally followed the interdisciplinary approach for renal transplant recipients with severe pneumonia in our ICU, although lacking the support of randomized controlled trials [4]. Rehabilitation after CIP/CIM is incomplete and often frustratingly slow, and many patients develop complications in an agonal state [11]. No specific therapy is available, but preventive and supportive measures can be beneficial in managing CIP/CIM. Early and aggressive treatment of the underlying disease is the fundamental strategy to prevent and treat CIP/CIM. Supportive measures include aggressive treatment of sepsis, reduction of the dosage and duration of neuromuscular blocking agents and corticosteroids, early rehabilitation, early enteral feeding, and anti-oxidant therapy [16]. Studies have suggested that physical therapy may help prevent CIP/CIM or reduce their severity. Although no definitive recommendations regarding physical therapy for CIP/CIM exist, an intensive physical therapy protocol might facilitate the recovery process in patients with CIP/CIM [17-19]. In our case, the patient experienced acute quadriplegia and subsequent rapid resolution after the aforementioned management strategies were used.

## Conclusion

CIP/CIM are complex syndromes that develop in critically ill patients. These syndromes should be diagnosed at an early stage. Treating the underlying disease, controlling the risk factors and initiating long-term rehabilitation might be beneficial to these patients.

## References

[1] Iwashyna TJ, Ely EW, Smith DM, Langa KM (2010). Long-term cognitive impairment and functional disability among survivors of severe sepsis. JAMA.

[2] Herridge MS, Cheung AM, Tansey CM, Matte-Martyn A, Diaz-Granados N, Al-Saidi F (2003). One-year outcomes in survivors of the acute respiratory distress syndrome. N Engl J Med.

[3] Gheith O, Al Otaibi T, Halim M, Said T, Nair P, Balaha M (2012). Successful management of critical illness polyneuropathy and myopathy in renal transplant recipients. Exp Clin Transplant.

[4] Tu GW, Ju MJ, Zheng YJ, Zhu DM, Xu M, Rong RM (2014). An interdisciplinary approach for renal transplant recipients with severe pneumonia: a single ICU experience. Intensive Care Med.

[5] Batt J, Dos SC, Cameron JI, Herridge MS (2013). Intensive care unit-acquired weakness: clinical phenotypes and molecular mechanisms. Am J Respir Crit Care Med.

[6] Derde S, Hermans G, Derese I, Guiza F, Hedstrom Y, Wouters PJ (2012). Muscle atrophy and preferential loss of myosin in prolonged critically ill patients. Crit Care Med.

[7] De Jonghe B, Sharshar T, Lefaucheur JP, Authier FJ, Durand-Zaleski I, Boussarsar M (2002). Paresis acquired in the intensive care unit: a prospective multicenter study. JAMA.

[8] Levine S, Nguyen T, Taylor N, Friscia ME, Budak MT, Rothenberg P (2008). Rapid disuse atrophy of diaphragm fibers in mechanically ventilated humans. N Engl J Med.

[9] Jaber S, Petrof BJ, Jung B, Chanques G, Berthet JP, Rabuel C (2011). Rapidly progressive diaphragmatic weakness and injury during mechanical ventilation in humans. Am J Respir Crit Care Med.

[10] Segredo V, Caldwell JE, Matthay MA, Sharma ML, Gruenke LD, Miller RD (1992). Persistent paralysis in critically ill patients after long-term administration of vecuronium. N Engl J Med.

[11] Kress JP, Hall JB (2014). ICU-acquired weakness and recovery from critical illness. N Engl J Med.

[12] Lacomis D, Zochodne DW, Bird SJ (2000). Critical illness myopathy. Muscle Nerve.

[13] Latronico N, Fenzi F, Recupero D, Guarneri B, Tomelleri G, Tonin P (1996). Critical illness myopathy and neuropathy. Lancet.

[14] Bednarik J, Lukas Z, Vondracek P (2003). Critical illness polyneuromyopathy: the electrophysiological components of a complex entity. Intensive Care Med.

[15] Zhou L, Zhao CB, Zhu WH, Xi JY, Lu J, Luo SS (2011). Clinical and pathological characteristics of steroid myopathy. Chin J Clin Neurosci.

[16] Eikermann M, Latronico N (2013). What is new in prevention of muscle weakness in critically ill patients?. Intensive Care Med.

[17] Griffiths RD, Palmer TE, Helliwell T, MacLennan P, MacMillan RR (1995). Effect of passive stretching on the wasting of muscle in the critically ill. Nutrition.

[18] Yosef-Brauner O, Adi N, Ben Shahar T, Yehezkel E, Carmeli E (2015). Effect of physical therapy on muscle strength, respiratory muscles and functional parameters in patients with intensive care unit-acquired weakness. Clin Respir J.

[19] Hermans G, De Jonghe B, Bruyninckx F, Van den Berghe G (2014). Interventions for preventing critical illness polyneuropathy and critical illness myopathy. Cochrane Database Syst Rev.

